# Artificial optic-neural synapse for colored and color-mixed pattern recognition

**DOI:** 10.1038/s41467-018-07572-5

**Published:** 2018-11-30

**Authors:** Seunghwan Seo, Seo-Hyeon Jo, Sungho Kim, Jaewoo Shim, Seyong Oh, Jeong-Hoon Kim, Keun Heo, Jae-Woong Choi, Changhwan Choi, Saeroonter Oh, Duygu Kuzum, H.-S. Philip Wong, Jin-Hong Park

**Affiliations:** 1 Department of Electrical and Computer Engineering, Sungkyunkwan University, Suwon, 16419 Korea; 2 SKKU Advanced Institute of Nanotechnology (SAINT), Sungkyunkwan University, Suwon, 16417 Korea; 3 Division of Materials Science and Engineering, Hanyang University, Seoul, 04763 Korea; 4 Division of Electrical Engineering, Hanyang University, Ansan, 15588 Korea; 5 Department of Electrical and Computer Engineering, University of California, San Diego, San Diego, CA 92093 USA; 6 Department of Electrical Engineering, Stanford University, Stanford, CA 94305 USA

## Abstract

The priority of synaptic device researches has been given to prove the device potential for the emulation of synaptic dynamics and not to functionalize further synaptic devices for more complex learning. Here, we demonstrate an optic-neural synaptic device by implementing synaptic and optical-sensing functions together on *h*-BN/WSe_2_ heterostructure. This device mimics the colored and color-mixed pattern recognition capabilities of the human vision system when arranged in an optic-neural network. Our synaptic device demonstrates a close to linear weight update trajectory while providing a large number of stable conduction states with less than 1% variation per state. The device operates with low voltage spikes of 0.3 V and consumes only 66 fJ per spike. This consequently facilitates the demonstration of accurate and energy efficient colored and color-mixed pattern recognition. The work will be an important step toward neural networks that comprise neural sensing and training functions for more complex pattern recognition.

## Abstract (web summary)

Artificial neural networks can emulate the human vision because of their spike-based operation by employing memristors as synapses. Here, Seo et al. integrate synaptic and optical sensing functions in a single heterostructure, which enables accurate and energy-efficient recognition of colored patterns.

## Introduction

Since Mead^1^ performed the first trial to mimic the biological neural networks (BNNs) of the brain in the 1980s, extensive effort has been made to emulate BNNs by utilizing various synaptic devices^2–13^. In order to properly perform signal processing in BNNs, synaptic plasticity and its timing-dependent learning algorithm are two key computational parameters^14^. However, the scope of initial research on synaptic devices has been mainly focused on simply mimicking synaptic dynamics, such as long-term potentiation/depression (LTP/LTD)^15^, short-term plasticity^16^, and spike-timing-dependent plasticity (STDP), with filament-forming switching devices^2,3^ or phase change memory devices^4^ in array crossbar structures. Such synaptic devices benefit from superior integration capability and energy efficiency compared to the synaptic devices with lateral transistor structure. However, they suffer from nonlinear potentiation/depression characteristics^17^, small differences between conduction states^18^, and insufficient conductance states^19,20^. Organic^5^ and carbon nanotube^6^ transistors have recently shown their suitability for emulating of synaptic dynamics with better linear potentiation/depression characteristics and a larger number of usable conductance states (>500 distinct states), in addition to high energy efficiency (<10 pJ switching energy)^21^.

Beyond this device-level emulation of synaptic dynamics, synaptic devices have also been used to build artificial neural networks (ANNs). Subsequently, pattern recognition tasks^2–8^ have been verified by these ANNs, where winner-take-all^6^ and perceptron networks^3^ are usually applied. However, none of the previous studies have tried to functionalize devices beyond their synaptic functions, for example, by merging them with biometric sensing elements such as vision, auditory, and olfactory sensors. Current works have only demonstrated signal processing in the cerebral cortex with either binary^2,3^ or grayscale MNIST (Modified National Institute of Standards and Technology) datasets^6^. The functional integration of synaptic devices with biometric sensing elements is expected to provide new opportunities for the implementation of neural networks that comprise neural sensing and training functions, thereby enabling power-efficient pattern recognition task for complex (e.g., color-mixed and voice-mixed) patterns. In this article, we demonstrate an optic-neural synaptic (ONS) device that features synaptic and optical-sensing functions. This ONS device was fabricated on a van der Waals (vdW) heterostructure (*h*-BN/WSe_2_)^22,23^, which does not have interfacial defects and thereby allows modulating a number of interfacial traps for achieving the synaptic functionalities. Through an optic-neural network (ONN) formed by these ONS devices, the colored and color-mixed pattern recognition capability of the human vision system is emulated. In particular, our synaptic device is investigated and compared with other devices reported heretofore, in terms of weight update linearity, number of usable conduction states, stability of each state, and energy efficiency (see Supplementary Table 1).

## Results

### *h*-BN/WSe_2_ optic-neural synaptic device

**Fig. 1:**
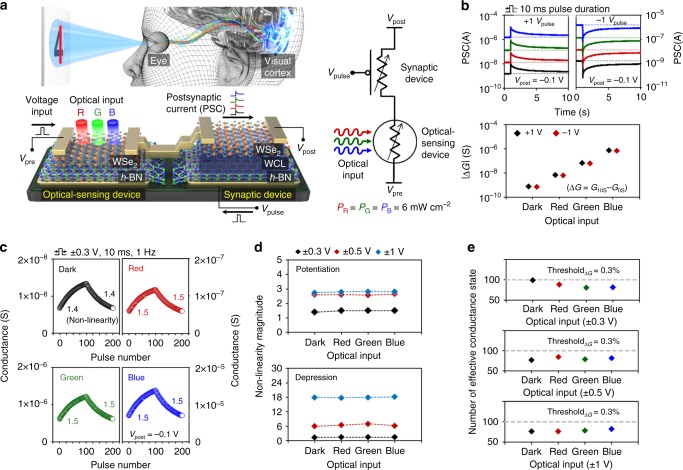
Integration of the *h*-BN/WSe_2_ optic-neural synaptic device. **a** Schematic of the human optic nerve system, the *h*-BN/WSe_2_ synaptic device integrated with *h*-BN/WSe_2_ photodetector, and the simplified electrical circuit for the ONS device. Here, the light sources were dot lasers with wavelengths of 655 nm (red), 532 nm (green), and 405 nm (blue) with a fixed power density (*P*) of 6 mW cm^−2^ for all wavelengths. **b** Excitatory and inhibitory postsynaptic current characteristics and extracted conductance changes of the *h*-BN/WSe_2_ ONS device under different light conditions (no light and RGB). **c** Long-term potentiation and depression curves under different light conditions, where the synaptic device is controlled using input pulses with an amplitude of 0.3 V. **d**, **e** Nonlinearity magnitude (**d**) and the number of effective conductance states (threshold_Δ*G*_ = 0.3%) (**e**), which were extracted for different wavelengths

As shown in Fig. 1, beyond the typical emulation of synaptic dynamics, we have fabricated an ONS device by integrating a synaptic device with an optical-sensing device on the same *h*-BN/WSe_2_ heterostructure. The operation of the vdW synaptic device is based on the trapping or de-trapping of electrons in the weight control layer (WCL) on *h*-BN, which modulates the WSe_2_ channel conductivity (weight of the synapse). The vdW synaptic device will be discussed in detail in the following subsections (see below). Here, the *h*-BN/WSe_2_ photodetector resistance is modulated as a function of the incident wavelength (see Fig. 1a). The additional analysis on the *h*-BN and WSe_2_ flakes is described in Supplementary Figure 1. The *h*-BN/WSe_2_ interface was confirmed to be clearly formed without polymer residues^24^, owing to our transfer method based on adhesion energy engineering (see Supplementary Figure 2). Because more optical absorption occurs at a shorter wavelength (see Supplementary Figure 3a), a shorter wavelength of light decreases the resistance of the optical-sensing device under a constant drain bias (see Supplementary Figure 3b, c). This reduction in resistance means that more carriers are generated in WSe_2_ so that the density of carriers trapped in the WCL increases. This subsequently allows the adjustment of the synaptic dynamic properties of the ONS device according to light wavelength conditions. Here, the optical-sensing device was fabricated on the same *h*-BN/WSe_2_ heterostructure where the synaptic device was formed^22,25^. This was done in order for the optical-sensing device to have the proper series resistance, which is comparable to that of the synaptic device, and to enable changing the synaptic properties. If the optical-sensing device has too much larger or smaller resistance value compared to the synaptic device, the resistance control in optical-sensing device by adjusting illumination condition will be ineffective to change the synaptic properties of the ONS device. To identify the synaptic dynamics of the ONS device under different light wavelength conditions, we investigated the device’s synaptic plasticity, the postsynaptic current (PSC), and LTP/LTD. The optical-sensing device was exposed to red (*λ* = 655 nm), green (*λ* = 532 nm), and blue (*λ* = 405 nm) lights with an optical power of 6 mW cm^−2^, and we then confirmed the PSC characteristics after applying ±1 V of voltage pulse (*V*_pulse_) to the synaptic device (see Fig. 1b and Supplementary Figure 4 for the measurement set-up). When exposed to a light with shorter wavelength, the magnitude of the synaptic current increased from 1.55 nA to 2.29 μA each, where the extracted conductance change (Δ*G*) also presented a significant increase from 0.78 nS to 0.74 μS by three orders of magnitude. As shown in Fig. 1c, the LTP/LTD curves were distributed in different conductance regions according to the light wavelength, but they maintained the curved shape related to nonlinearity. If the conductance state varies too nonlinearly, similar to a square root function, additional periphery circuits are typically required to manage the wide range of conductance state changes^26,27^. In addition, a nonlinear conductance response imposes difficulty in achieving a sufficient number of usable conductance states because a voltage pulse disables the induction of a sufficiently large conductance difference in the saturated region. Here, we defined effective conductance states as those in which Δ*G* exceeds a certain percentage of *G*_max_/*G*_min_ (threshold_Δ*G*_ as described in detail in Supplementary Figure 5). Based on threshold_Δ*G*_, we excluded the pulse signals that induced states with small Δ*G* unimportant for neuromorphic computing, thus reducing power consumption during computation. For the quantitative analysis of the LTP/LTD characteristics of our ONS device, we extracted a nonlinearity^28^ (see Fig. 1d and Supplementary Figure 6) and the number of effective conductance states (see Fig. 1e). For case 1 with a pulse amplitude of ±0.3 V, the nonlinearity was maintained at the levels of 1.5 (potentiation)/1.5 (depression), regardless of the different light conditions, including a no-light condition. In the remaining pulse conditions with pulse amplitudes of ±0.5 V (case 2) and ±1 V (case 3), the nonlinearity (2.6/6 for case 2 and 2.7/18 for case 3) was higher than the values of case 1; however, they were also independent of the incident light wavelength. Moreover, the number of effective conductance states did not significantly change despite the different light conditions (82–99 for case 1, 75–82 for case 2, and 74–82 for case 3), where threshold_Δ*G*_ was set to 0.3%. Overall, we observed that (i) the conductance ranges in which the LTP/LTD synaptic characteristics were clearly different as a function of the illuminating wavelength; (ii) the LTP/LTD characteristics, such as the linearity and the number of effective conductance states that significantly affect the pattern recognition rate, were almost independent of the light wavelength. Because an optical power is another important information about the optical input, the ONS device eventually needs to be distinctly operated according to the power as well as the wavelength of the optical input. To account for environmental challenges in real-world applications, we exposed our devices to ambient conditions over an extended period of time (see Supplementary Figure 7). Even after 1 month under such conditions_,_ our device proved to be unaffected and worked properly, highlighting their potential for future applications.

### Characteristics of *h*-BN/WSe_2_ synaptic device

**Fig. 2:**
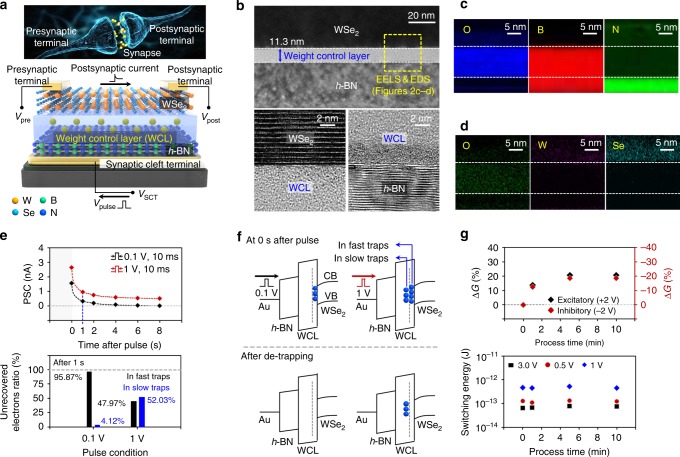
Structure and operating mechanism of the *h*-BN/WSe_2_ synaptic device. **a** Functional/structural/architectural comparison of biological synapse with our synthetic WSe_2_/WCL/*h*-BN synaptic device. **b** X-TEM image of the WSe_2_/WCL/*h*-BN structure, and the high-resolution images corresponding to the WSe_2_/WCL and WCL/*h*-BN interfaces. **c**, **d** EELS (**c**) and EDS (**d**) mapping images obtained on the cross-section of the WSe_2_/WCL/*h*-BN structure. **e** Current relaxation curves after pulse amplitudes of 0.1 V and 1 V, and contribution ratio from unrecovered electrons in fast traps and slow traps at 1 s after the pulse. **f** Illustration of energy band diagrams after pulse and after de-trapping of carriers in fast traps. **g** Change in postsynaptic conductance and the switching energy measured as a function of O_2_ plasma process time. Here, all the *V*_pulse_ were applied with a duration of 10 ms

The synaptic plasticity of the ONS device derives from the operation of the vdW synaptic device with the WCL. The first step toward achieving this synaptic device was to create a charge trapping layer on top of *h*-BN for adjustment of the WSe_2_ channel conductivity^29^. By forming the WCL on *h*-BN with an O_2_ plasma treatment, we implemented the vdW synaptic device, as shown in Fig. 2a. The hysteresis characteristic was observed in the current between the presynaptic and postsynaptic terminals, which was dependent on the voltage applied to the synaptic cleft terminal (*V*_SCT_). This occurs because charges trapped in the WCL partially screen *V*_SCT_ and thereby influence the current flow through the synaptic device (see Supplementary Figure 8). Figure 2b shows cross-sectional transmission electron microscopy (X-TEM) images taken near the WCL of the synaptic device. After the O_2_ plasma treatment was performed for 5 min, an 11.3 nm-thick WCL was observed on the *h*-BN surface region. In addition, as seen in Fig. 2c, high-resolution electron energy loss spectroscopy (EELS) and energy-dispersive X-ray spectroscopy (EDS) mapping analyses were performed to investigate the atomic compositions of the WSe_2_/WCL/*h*-BN region. In the WCL, signals related to O and B elements clearly appeared, but N, W, and Se signals were not present, indicating that the WCL consisted of the oxidized boron transformed from *h*-BN. As expected, this WCL was not observed in the EELS and EDS mapping images for the control sample that was not subjected to the O_2_ plasma treatment (refer to Supplementary Figure 9). To analyze the dynamic nature of the conductivity (see Fig. 2e), voltage pulses with 0.1 and 1 V amplitudes were applied to the synaptic device. In the case where the voltage pulse was 0.1 V, the transient electrons trapped in fast traps recovered, and the abruptly increased current returned to its initial value after 8 s, resulting in no persistent Δ*G*. On the other hand, when a pulse with 1 V amplitude was applied, a positive Δ*G* occurred because electrons were captured and held in the traps deeper inside the WCL. Calculation of the trap density in the WCL gave a value of approximately 7.4 × 10^−17^ cm^−3^. When extrapolated to current memory cell sizes, the number of trapped electrons was on the same order of electrons stored on a floating gate of a flash memory cell (see Supplementary Figure 10) (http://www.itrs2.net/). The Fig. 2e shows the ratio of the remaining charges in fast traps and slow traps at 1 s after the pulse, according to the pulse amplitude. When a pulse with higher amplitude was applied, the contribution of slow traps to the increase in PSC increased noticeably, consequently exceeding that of fast traps. As shown in Fig. 2f, by applying a pulse with higher amplitude, more charges are expected to be trapped in slow traps that will not recover at room temperature, which subsequently changes the synaptic conductance of WSe_2_. We also note that the contribution of fast traps to the conductance change diminishes as time elapses after the pulse (see Supplementary Figure 11). The magnitude of Δ*G* increased until the O_2_ process time reached the 5 min point and then saturated beyond that time, where the |Δ*G*| values were similar in both excitatory and inhibitory synaptic responses. We also estimated the switching energy of the synaptic device as a function of the O_2_ plasma process time (see Fig. 2g). Regardless of the process time related to the amount of traps in the WCL, the switching energy was approximately 66 fJ (at 0.3 V of presynaptic pulse) and it increased to 532 fJ (at 1 V of pulse), which is comparable to those of previously reported synaptic devices (see Supplementary Table 1b). Δ*G* is a function of the amplitude and duration of the presynaptic pulse for the weight update, such that the dissipated energy per event can be determined by *P* = *I* × *V* × *t*_duration_^5^.

**Fig. 3:**
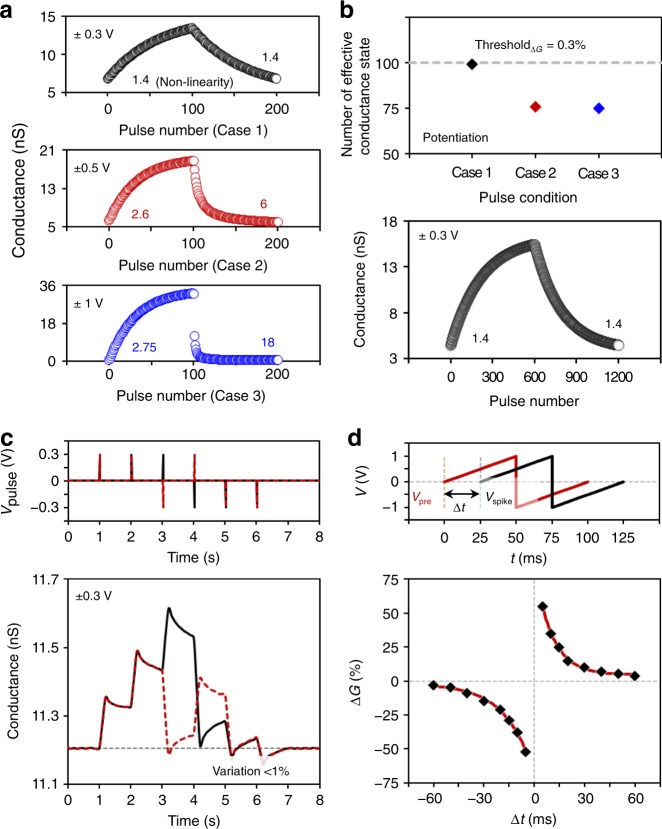
Synaptic plasticity characterization of *h*-BN/WSe_2_ synaptic device. **a** Long-term potentiation and depression characteristics by different input pulses with an amplitude of 0.3 V, 0.5 V, or 1 V. **b** Number of effective conductance states for the three cases with a threshold_Δ*G*_ = 0.3%, and LTP/LTD curves when 600 pulses are applied in each potentiation and depression. **c** Stability of conductance states with below 1% variation. **d** Spike-timing-dependent plasticity behavior obtained in the *h*-BN/WSe_2_ synaptic device. The pre-spike and post-spike voltages are applied to the presynaptic and SCT, respectively

For neuromorphic computing based on deep neural networks (DNNs) with the well-studied back-propagation learning algorithm^30^, as mentioned above, synaptic devices are required to have the following characteristics: linear conductance responses^17,19,31^, a sufficient number of effective conductance states^19,20,31^, and high stability in each state. Here, we thoroughly investigated the vdW synaptic device to identify various synaptic dynamics including LTP/LTD and STDP. Figure 3a shows the LTP/LTD characteristic curves for the synaptic device according to three pulse conditions: ±0.3 V (case 1), ±0.5 V (case 2), and ±1 V (case 3). When applying a lower amplitude pulse, the conductance was changed more linearly, subsequently presenting a low nonlinearity of 1.4 (case 1). Figure 3b shows that 99 (case 1), 75 (case 2), and 74 (case 3) conductance states were usable when threshold_Δ*G*_ was set to 0.3%. Additionally, to confirm if this synaptic device can achieve the number of conductance states that were typically demanded for inference of DNNs (6−8 bits = 64−256 states)^32,33^, we applied excitatory and inhibitory pulses 600 times each (see Fig. 3b). As a result, the nonlinearity was approximately 1.4, and the number of effective conductance states was 599 under a 0.3% threshold_Δ*G*_. Each conductance state was very stable, presenting a very small variation of below 1% even after the state was changed many times by the excitatory and inhibitory pulses. This can be explained by a similar number of electrons which are trapped and de-trapped in the *h*-BN WCL by applying symmetric positive and negative pulses. In the Supplementary Figure 12, we additionally discuss the long-term variation of conductance states with respect to the various pulse combinations. In Fig. 3d, we also confirm the STDP behavior of our vdW synaptic device, indicating that this device can be applied to spiking neural networks with the STDP learning algorithm^14^. Biological synapses potentiate if a presynaptic spike precedes a postsynaptic spike, and depress if a postsynaptic spike precedes a presynaptic spike^34^. Likewise, in our synaptic device, conductance increased or decreased (Δ*G*/*G* > 0 or Δ*G*/*G* < 0) according to the relative difference between the presynaptic spike (*V*_pre_) and the SCT spike (*V*_SCT_) (Δ*t* > 0 or Δ*t* < 0).

### Colored and color-mixed pattern recognition

**Fig. 4:**
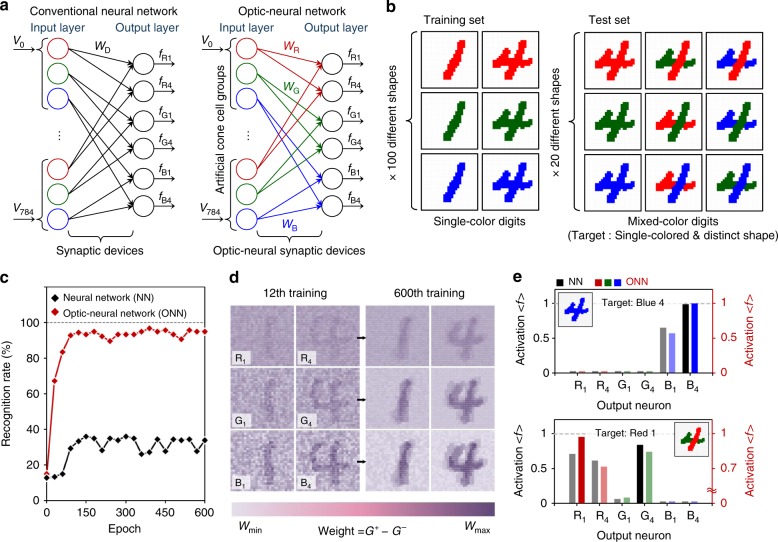
Colored and color-mixed pattern recognition based on an artificial optic-neural network. **a** Developed ONN for recognition of 28 × 28 RGB-colored images. **b** Examples of the training and the testing datasets consisting of single-colored and color-mixed numeric pattern images, respectively. **c** Recognition rate as a function of number of training epochs. **d** Weight mapping images after the 12th and 600th training epoch. **e** Activation values of each output neuron in cases of a single-colored number (blue 4) and a color-mixed number (red/green-mixed 4) after the 600th training epoch

Following the integration of our synaptic device and optical-sensing device on the *h*-BN/WSe_2_ heterostructure, we developed an artificial ONN using the extracted device parameters and a simple perceptron network model^35^, finally applying the ONN to the colored and color-mixed pattern recognition tasks. As shown in Fig. 4a, two separate neural networks were developed for recognizing the target color number from the complex mixed-color numerical digits (similar to a color-blindness test). Here, the input layer of the conventional neural network (NN) consisted of the neuron array with the color-filtering function to emulate the biological cone cells of the human vision system, but the synaptic connections were formed without the optical-sensing function. In contrast, in the ONN, an optical-sensing function was added to the synaptic connection. Our artificial cone cell group consisted of 3 neurons, and the groups formed a 28 × 28 array, where each cell in the group responded differently to the wavelengths of visible red (R), green (G), and blue (B) light as human eyes do, subsequently inducing different synaptic dynamics. The input signals for both NN and ONN were determined as 1 V (for R), 0.5 V (for G), and 0.3 V (for B) by considering the color. Neurons in each cone cell are fully connected to 2 dedicated classifying neurons (“1” and “4”), resulting in a connection with 6 classifying neurons at the output layer. In particular, the black weight connections in the NN represent the synaptic devices with the LTP/LTD characteristic under dark condition, and the connections expressed by RGB (red, green, and blue) color lines in the ONN indicate the synaptic devices with the LTP/LTD characteristics in different R/G/B conductance ranges. The MNIST dataset^36^ was used for the pattern recognition tasks, in which the original image size (28 × 28) was preserved, but a simple modification was made to generate color-mixed numerical patterns (see Supplementary Figure 13). The objective of this pattern recognition task was to recognize the target numbers from the color-mixed test images (similar to a color-blindness test), where the target numbers are buried in the color-mixed patterns. To train the networks, as seen in Fig. 4b, we prepared 6 different training datasets with 100 training images for each dataset (600 images in total) for RGB-colored numbers 1 and 4. We also prepared 9 test datasets with 20 different test images for each dataset (180 images in total) for the pattern recognition test. The 600 different training images were applied to the networks at every training epoch, and we finished the training phase at 600 epochs. At every epoch, the recognition rates for both networks were estimated using 180 test images. The rate for the ONN exceeded 90% after the 50th epoch and then stabilized (see Fig. 4c). In contrast, the recognition rate for the NN, which was composed of synaptic devices without the optical-sensing function, was below 40%. As the training epochs increased, the synaptic weight values were further optimized for RGB-color-mixed numerical pattern recognition. To confirm this weight optimization, we reconstructed and visualized the synaptic weight values after the 12th and 600th training epochs (see Fig. 4d). The synaptic weights created for the blue patterns became richer than those of the other colors did because the ONS device most strongly responded to blue light input; this indicates that the ONN successfully recognized different-colored numerical patterns (RGB 1 or 4). Finally, the recognition tests for color-mixed digit patterns were demonstrated for both networks with the well-trained weight values after the 600th training epoch, and the corresponding results are provided in Fig. 4e. In the case of the blue-colored number 4, both networks easily succeeded in recognizing the target number by providing the highest activation scores at the B4 classifying (output) neurons. However, for the complex color-mixed number (red/green-mixed 4), the R1 classifying neuron in the ONN produced the highest activation score of 0.98, while the highest value of 0.83 was obtained from the G4 classifying neuron in the NN.

## Discussion

We demonstrate an ONS device with synaptic and optical-sensing functions by integrating a synaptic device and an optical-sensing device on a vdW heterostructure (*h*-BN/WSe_2_). Our ONS device exhibit various synaptic dynamics like LTP, LTD, and STDP according to the light conditions (red (*λ* = 655 nm), green (*λ* = 532 nm), and blue (*λ* = 405 nm)), while maintaining its synaptic plasticity (linearity and number of effective conductance states), where the LTP/LTD characteristics are distributed in different conductance regions. In the vdW synaptic device, the conductance related to the synaptic weight is adjusted by the charges trapped in the WCL that is formed on *h*-BN by O_2_ plasma treatment. This vdW synaptic device in particular shows good linearity with nonlinearity = 1.4/1.4 for weight increase/decrease, a number of effective conductance states about 599 at threshold_Δ*G*_ = 0.3%, and very small variation below 1% after the random state changes by excitatory and inhibitory pulses. These excellent synaptic properties clearly highlight the potential of our vdW-based ONS device for building highly accurate neuromorphic ANNs. Following in-depth study and characterization of the vdW ONS device, we develop an ONN that emulates the colored and color-mixed pattern recognition capability of the human vision system. With this ONN we achieve >90% recognition rate for color-pattern recognition task, which is similar to a color-blindness test. With this research, where we co-integrate synaptic and biometric sensing elements on the same technology stack, we provide the foundation for future work towards building neural networks that comprise sensing and training functionalities for highly integrated complex pattern recognition tasks.

## Methods

### Fabrication and characterization of the synaptic device

The individual control electrodes (line shaped with width of 20 μm) were patterned on the 90 nm-thick SiO_2_ oxide layer on heavily boron-doped Si substrate using an optical lithography process, followed by 10 nm-thick Ti and 30 nm-thick Au deposition processes via an e-beam evaporator. The *h*-BN flake was then transferred onto the control electrode by our residue-free transfer method based on adhesion energy engineering (see Supplementary Figure 2). The O_2_ plasma treatment process was carried out on the *h*-BN flake by a plasma machine (Miniplasma Cube, PLSMART). To stabilize the chamber conditions, O_2_ gas flowed for 2 min before the O_2_ plasma treatment. The treatment conditions are as follows: reactive ion etcher power (20 W, 100 W), plasma pressure (470 mTorr), and O_2_ flow rate (5 sccm), treatment time (1, 5, and 10 min). A WSe_2_ flake was then transferred onto the WCL/*h*-BN by using the same transfer method. The postsynaptic and presynaptic electrodes (distance between two electrodes and width of the electrodes were 5 μm) were patterned on the WSe_2_/WCL/*h*-BN sample by an optical lithography process, followed by 10 nm-thick Pt and 50 nm-thick Au deposition processes via an e-beam evaporator (see Supplementary Figure 1, optical image of the synaptic device). For the structural and elemental analyses of the WSe_2_/WCL/*h*-BN region, the X-TEM (JEM ARM 200F) and EELS/EDS (GIF quantum ER, 200 keV) measurements were employed. The fabricated WSe_2_/WCL/*h*-BN synaptic devices were electrically analyzed in terms of current with respect to the control voltage by an HP 4155A semiconductor parameter analyzer.

### Fabrication and characterization of the optical-sensing device

The *h*-BN flake was mechanically exfoliated and transferred onto the 90 nm-thick SiO_2_ layer on heavily boron-doped Si substrate via adhesive tape (224SPV, Nitto). The remaining adhesive tape residue was removed by immersing the samples in an acetone bath for 1 h. The WSe_2_ flake was then transferred to the *h*-BN/SiO_2_/Si substrate by the residue-free transfer method. The electrodes with 5 μm spacing were patterned on the *h*-BN/WSe_2_ sample by an optical lithography process, which were respectively grounded and connected to the presynaptic terminal of the vdW synaptic device, then followed by 10 nm-thick Pt (for p-type channel) and 50 nm-thick Au deposition processes via an e-beam evaporator (see Supplementary Figure 1, optical image of the optical-sensing device). The *h*-BN/WSe_2_ optical-sensing devices were investigated by electrical measurements (current with respect to control voltage, *I*–*V*) using an HP 4155A semiconductor parameter analyzer under both dark and illuminated conditions. The light sources were dot lasers with wavelengths of 655 nm (red), 532 nm (green), and 405 nm (blue), and an optical power of 6 mW cm^−2^.

### Pattern recognition task

After integrating the synaptic device with the optical-sensing device on the *h*-BN/WSe_2_ heterostructure, in order to confirm the learning capability of our ONS device, we designed neural networks based on a single-layer perceptron model using the extracted ONS device parameters, such as the nonlinearity values (at input signal pulse = ± 0.3 V) and the number of effective conductance states (at threshold_Δ*G*_ = 0.3%). We then investigated the recognition rates for the colored MNIST numerical patterns. When the input voltage signals corresponding to the RGB colors (red = 1 V, green = 0.5 V, blue = 0.3 V) were applied to the input neurons, currents were obtained at output neurons by a matrix product of the input signal and the synaptic weight (*W*) values. Here, the synaptic weight is defined by the difference between the conductance values of the two ONS devices (*W* = *G*^+^ − *G*^−^). The currents at the output neurons were transformed by a sigmoid activation function, resulting in output neuron signals (*f*). Based on the delta value (*δ*), which is the difference between the output neuron signals (*f*) and the label values (*k*) for the input patterns (*δ* = *k* − *f*), the synaptic weight was determined to be potentiated or depressed. If *δ* > 0 (potentiation phase), then *G*^+^ is increased and *G*^−^ is simultaneously decreased, thereby increasing the synaptic weight (*W*↑ = *G*^+^↑ − *G*^−^↓). In the depression phase (*δ* < 0), *G*^+^ and *G*^−^ are decreased and increased, respectively, at the same time (*W*↓ = *G*^+^↓ − *G*↑). These conductance changes (∆*G*^+^ or ∆*G*^−^) were determined by the following equations:

1 $$G_{{\mathrm{n}} + 1} = G_{\mathrm{n}} + {\mathrm{\Delta }}G_{\mathrm{P}} = G_{\mathrm{n}} + {\mathrm{\alpha }}_{\mathrm{P}}e^{ - \beta _{\mathrm{P}}\frac{{G_{\mathrm{n}} - G_{{\mathrm{min}}}}}{{G_{{\mathrm{max}}} - G_{{\mathrm{min}}}}}}({\mathrm{\Delta }}G^ + \,{\mathrm{or\Delta }}G^ - > 0,G^ + \,{\mathrm{or}}\,G^ - \uparrow )...,$$

2 $$G_{{\mathrm{n}} + 1} = G_{\mathrm{n}} + {\mathrm{\Delta }}G_{\mathrm{D}} = G_{\mathrm{n}} - {\mathrm{\alpha }}_{\mathrm{D}}e^{ - \beta _{\mathrm{D}}\frac{{G_{{\mathrm{max}}} - G_{\mathrm{n}}}}{{G_{{\mathrm{max}}} - G_{{\mathrm{min}}}}}}({\mathrm{\Delta }}G^ + \,{\mathrm{or\Delta }}G^ - < 0,G^ + \,{\mathrm{or}}\,G^ - \downarrow )...$$

In these equations, *G*_*n*+1_ and *G*_*n*_ denote the synaptic conductance when the *n* + 1th and *n*th pulses are applied, and parameters *α* and *β* are the conductance change amount and the nonlinearity, respectively (see Supplementary Table 2). The above pattern recognition processing was implemented with MATLAB.

### Code availability

Code from this study (MATLAB scripts) is available from the corresponding author upon request.

## Electronic supplementary material

Supplementary Information

## Data Availability

The data that support the findings of this study are available from the corresponding author upon request.
