# Supplementary material for: Artificial optic-neural synapse for colored and color-mixed pattern recognition
Source: Nat Commun. 2018 Nov 30;9:5106. doi: 10.1038/s41467-018-07572-5 (PMC6269540; doi:10.1038/s41467-018-07572-5)
Supplement: Supplementary file 1 — Supplementary Information [file 41467_2018_7572_MOESM1_ESM.pdf]

# Supplementary information:

## Artificial Optic-Neural Synapse for Colored and Color-Mixed Pattern Recognition

**Seo et al.**

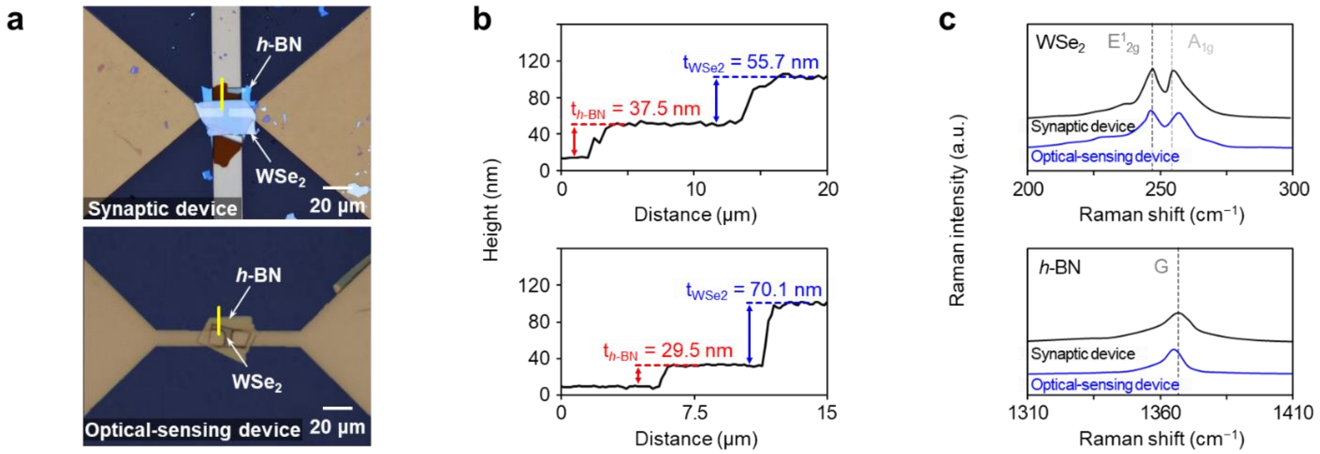


**Supplementary Figure 1 | Characterization of *h*-BN/$WSe_2$ heterostructure. a,** Optical image of synaptic device and optical-sensing device fabricated on *h*-BN/$WSe_2$ heterostructure. **b**, Thickness of the *h*-BN and $WSe_2$ flakes measured by atomic force microscopy, along the yellow lines as marked in **a**. **c**, Raman spectra on the $WSe_2$ and *h*-BN regions. In the $WSe_2$ flakes, two conventional Raman peaks ($E^1_{2g}$ and $A_{1g}$) are observed near 245 and 255 $cm^{-1}$, where the peaks indicate the in- and out-of-plane vibrations for bulk $WSe_2$, respectively. In the *h*-BN flakes, the Raman peak ($E^1_{2g}$) near 1366 $cm^{-1}$ indicates the in-plane vibration for bulk *h*-BN.

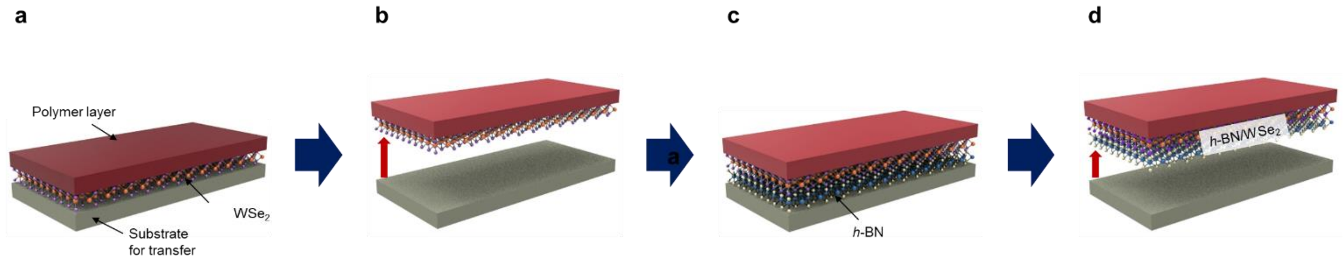


**Supplementary Figure 2 | Residue-free transfer method based on adhesion energy engineering. a,** The $WSe_2$ flake was exfoliated on a substrate. **b**, Next, the $WSe_2$ flake was picked up by using a polymer layer (e.g., PMMA, PPC). Because the adhesion energy between the $WSe_2$ flake and the polymer layer is stronger than that between the $WSe_2$ flake and the substrate, the $WSe_2$ flake easily attaches to the bottom of the polymer layer and is picked up. **c**, **d**, The *h*-BN flake was prepared on a substrate and was picked up using the same transfer method. By using this residue-free transfer method based on adhesion energy, a clean *h*-BN/$WSe_2$ interface without polymer residues was formed.

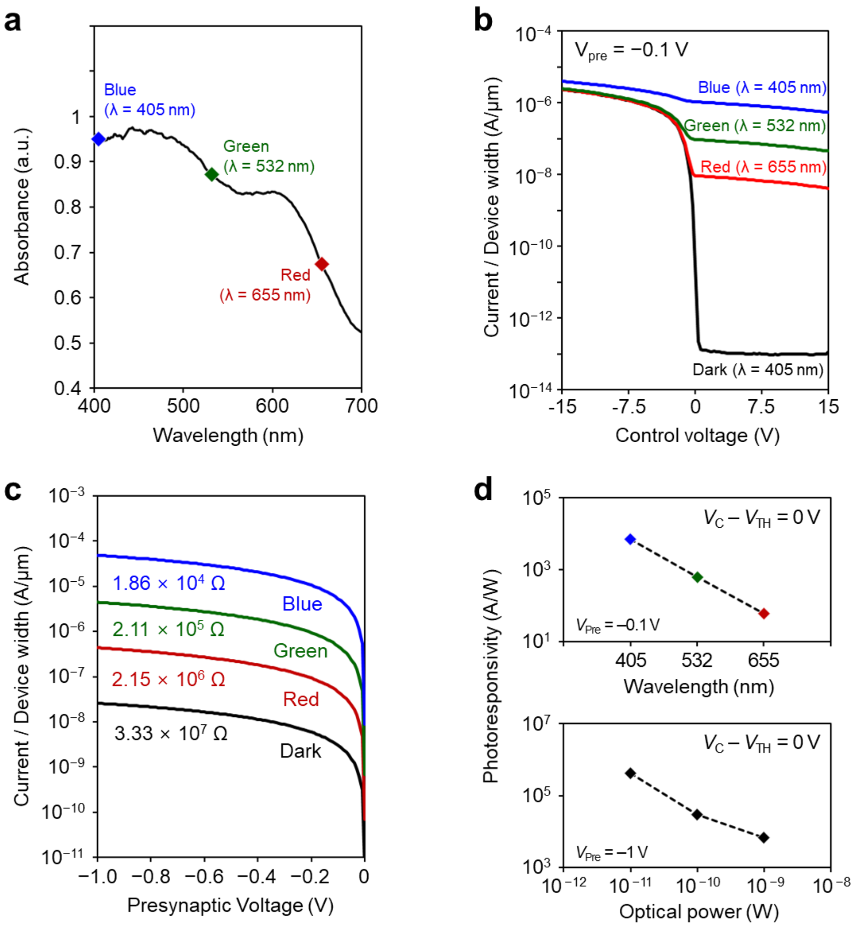


**Supplementary Figure 3 | Absorbance and current of the vdW optical-sensing device at different lighting conditions. a,** Absorbance vs. wavelength (λ). **b,** Current per device width with respect to control voltage ($V_C$). **c,** Current per device width with respect to presynaptic voltage ($V_{Pre}$) **d,** Photoresponsivity of the optical-sensing device with respect to the wavelength of light and with respect to the optical power.

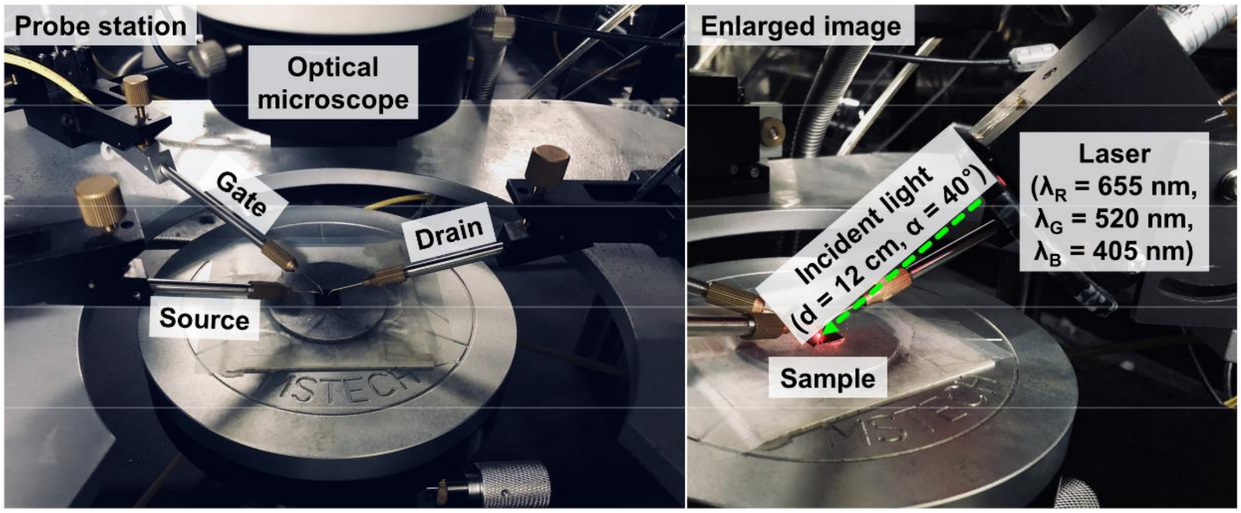


**Supplementary Figure 4 | Measurement set-up for optic neural synaptic device.** To minimize the laser intensity variation, a laser source was fixed in a holder. The distance between the laser source and the sample was 12 cm, and the light incident angle was 40 degrees.

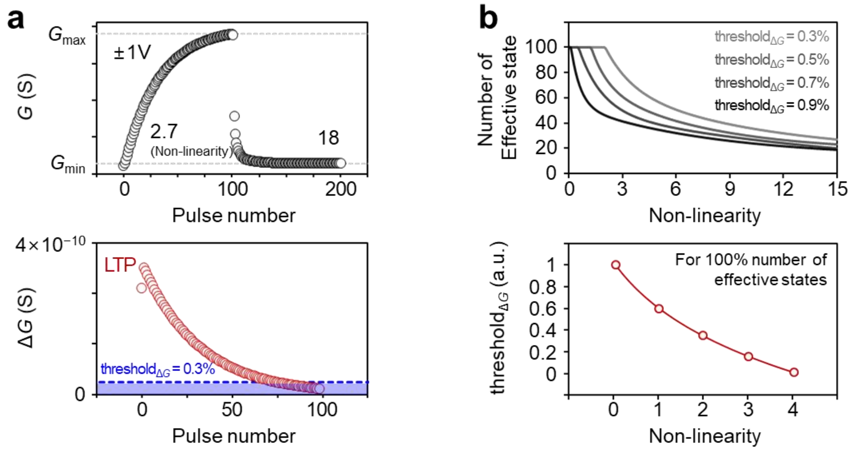


**Supplementary Figure 5 | Number of effective conductance states. a,** LTP curves and $\Delta G$ extractions with respect to the number of pulses with +1 V amplitude. **b**, Number of effective conductance states at various threshold$_{\Delta G}$ values and the minimum threshold$_{\Delta G}$ value to achieve full effective conductance states with respect to the nonlinearity.

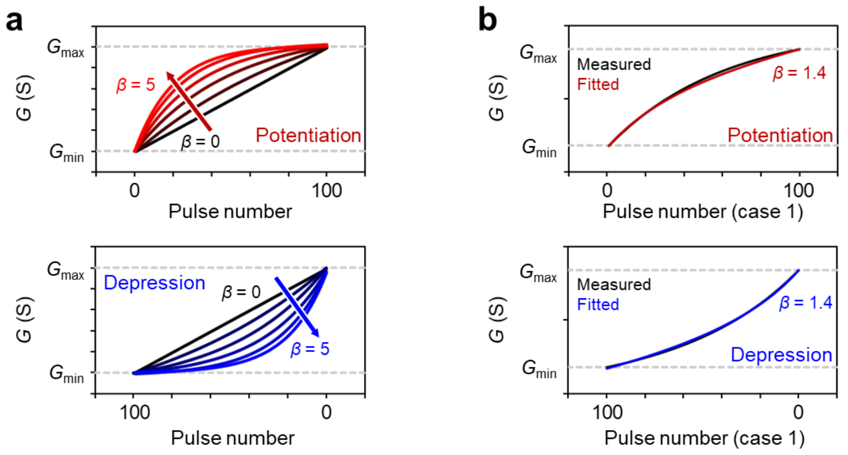


**Supplementary Figure 6 | Nonlinearity analysis of LTP/LTD curves. a,** Long-term potentiation and long-term depression with respect to the nonlinearity, β, ranging from 0 to 5. **b,** Fitting curves of both LTP and LTD using the extracted parameters given in Supplementary Table 2 (see below) at β = 1.4

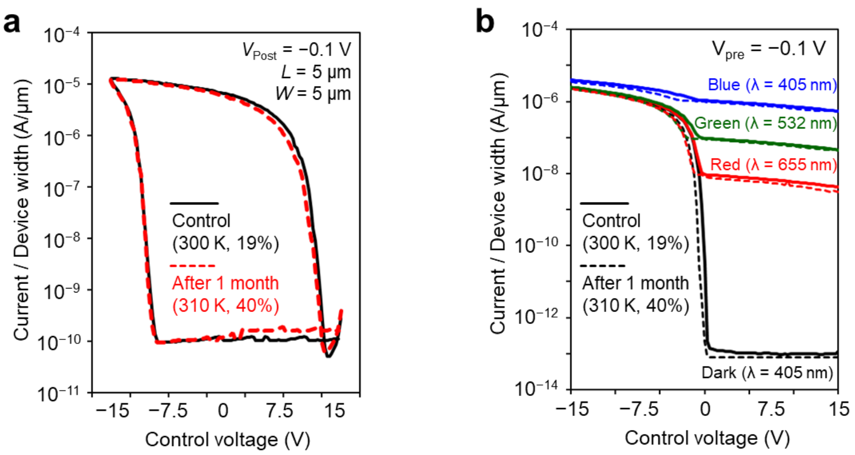


**Supplementary Figure 7 | Long-term stability test of synaptic and optical-sensing devices. a, b,** Long-term stability test of synaptic device and optical-sensing device. Here, we measured the *I-V* characteristic curves of synaptic and optical-sensing devices right after fabrication and after 1 month in air (Condition 1: Temperature = 300 K, relative humidity = 19%; Condition 2: Te mperature = 310 K, relative humidity = 40%).

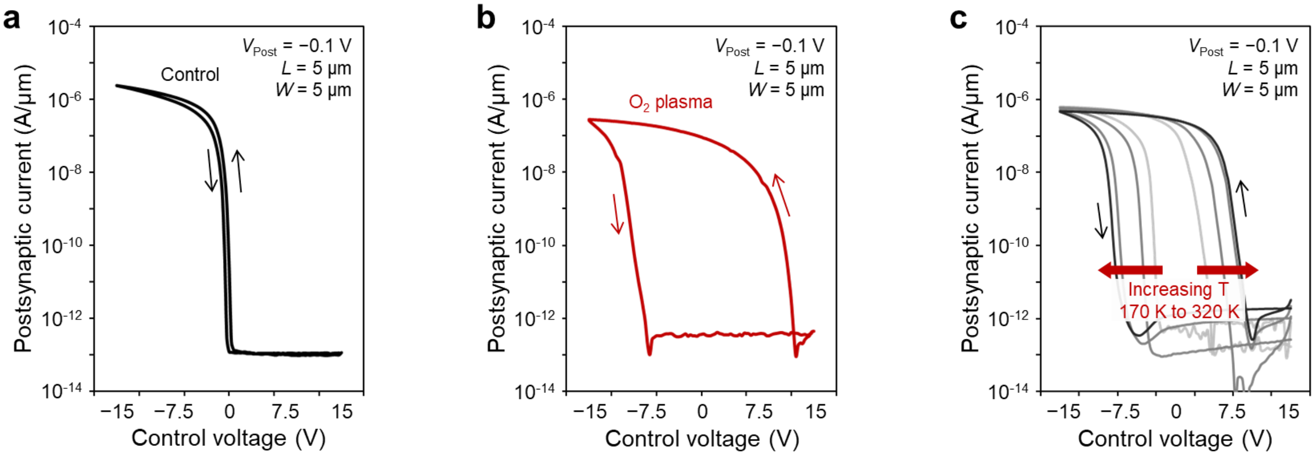


**Supplementary Figure 8 | Electrical transfer curves of vdW synaptic devices. a,** Synaptic device without weight control layer (WCL). **b,** Synaptic device with WCL formed by $O_2$ plasma treatment. **c,** $I_{Post}$-$V_C$ curves with respect to temperature (170 K, 220 K, 270 K, and 320 K).

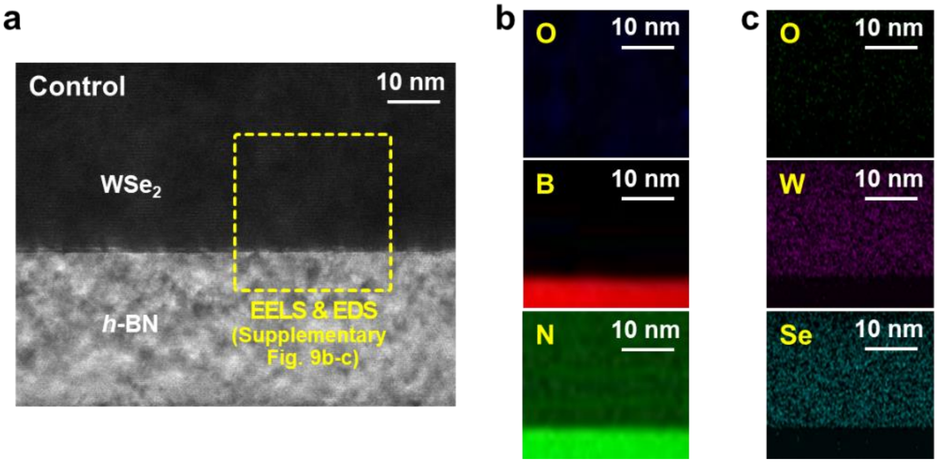


**Supplementary Figure 9 | High resolution X-TEM images, EELS, and EDS mapping analyses. a,** High-resolution cross-sectional transmission electron microscopy (X-TEM) image. **b,** Electron energy loss spectroscopy (EELS) mapping images. **c,** Energy-dispersive X-ray spectroscopy (EDS) mapping images

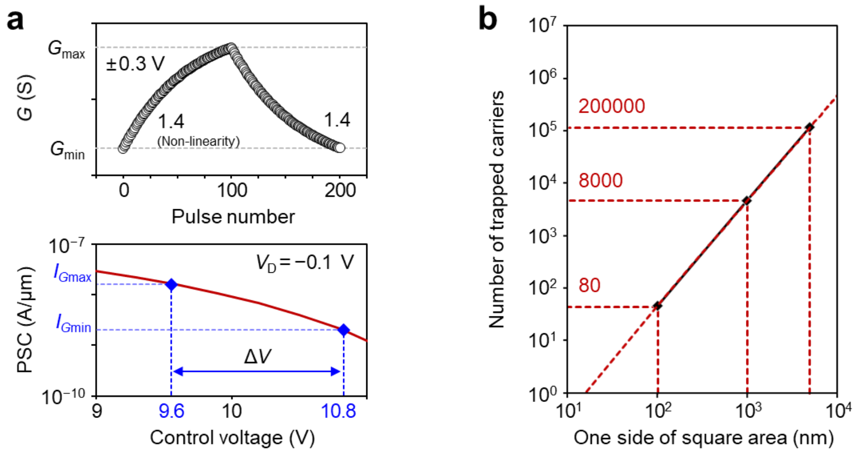


**Supplementary Figure 10 | Approximate uniform trap density in WCL. a,** Control voltage range for ensuring $G_{min}$ to $G_{max}$. **b,** Number of trapped carriers w.r.t one side of the square area

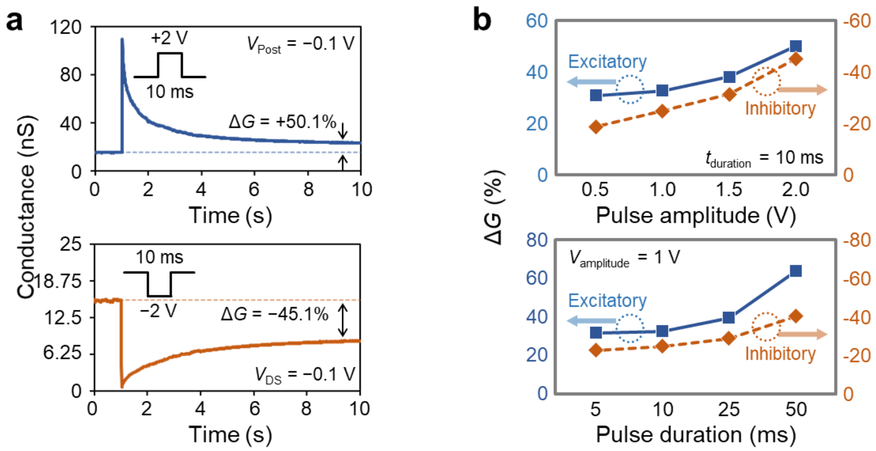

**Supplementary Figure 11 | Post-synaptic current analysis under different pulse conditions. a,** Conductance trajectories of excitatory post-synaptic current (EPSC) and inhibitory post-synaptic current (IPSC) when applying pulses with 10 ms width and 2 V amplitude (positive for EPSC and negative for IPSC) to the synaptic cleft terminal (SCT). **b,** $\Delta G$ extractions under different pulse amplitude and duration for both EPSC and IPSC

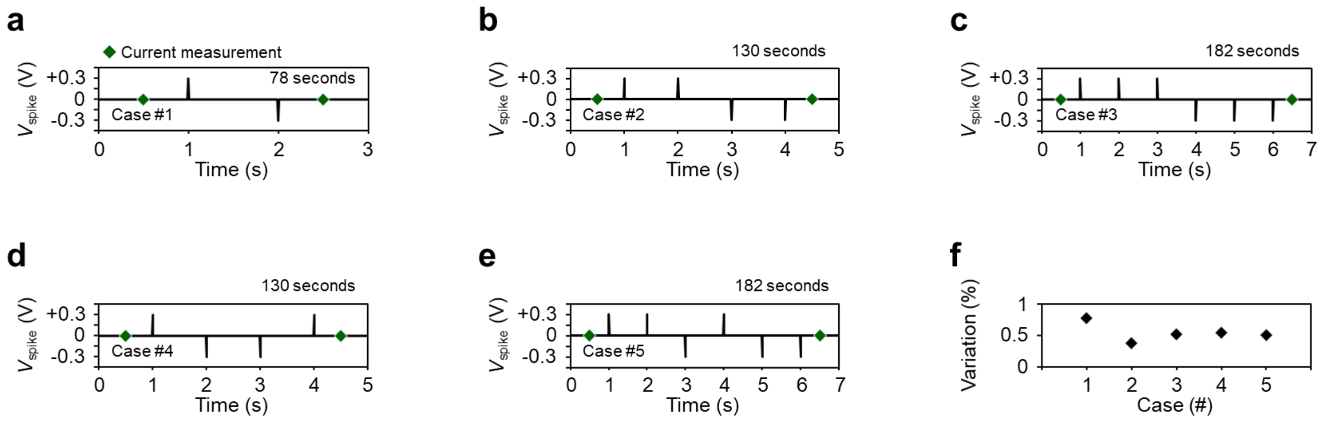


**Supplementary Figure 12 | Conductance state variation with respect to various pulse combinations. a, b, c, d, e,** Various combinations of pulses with ±0.3 V amplitude, which were applied for 78 - 182 seconds (26 times in each case). **f,** The average variations for the five cases, which were 0.78%, 0.38%, 0.52%, 0.54%, 0.51%, respectively.

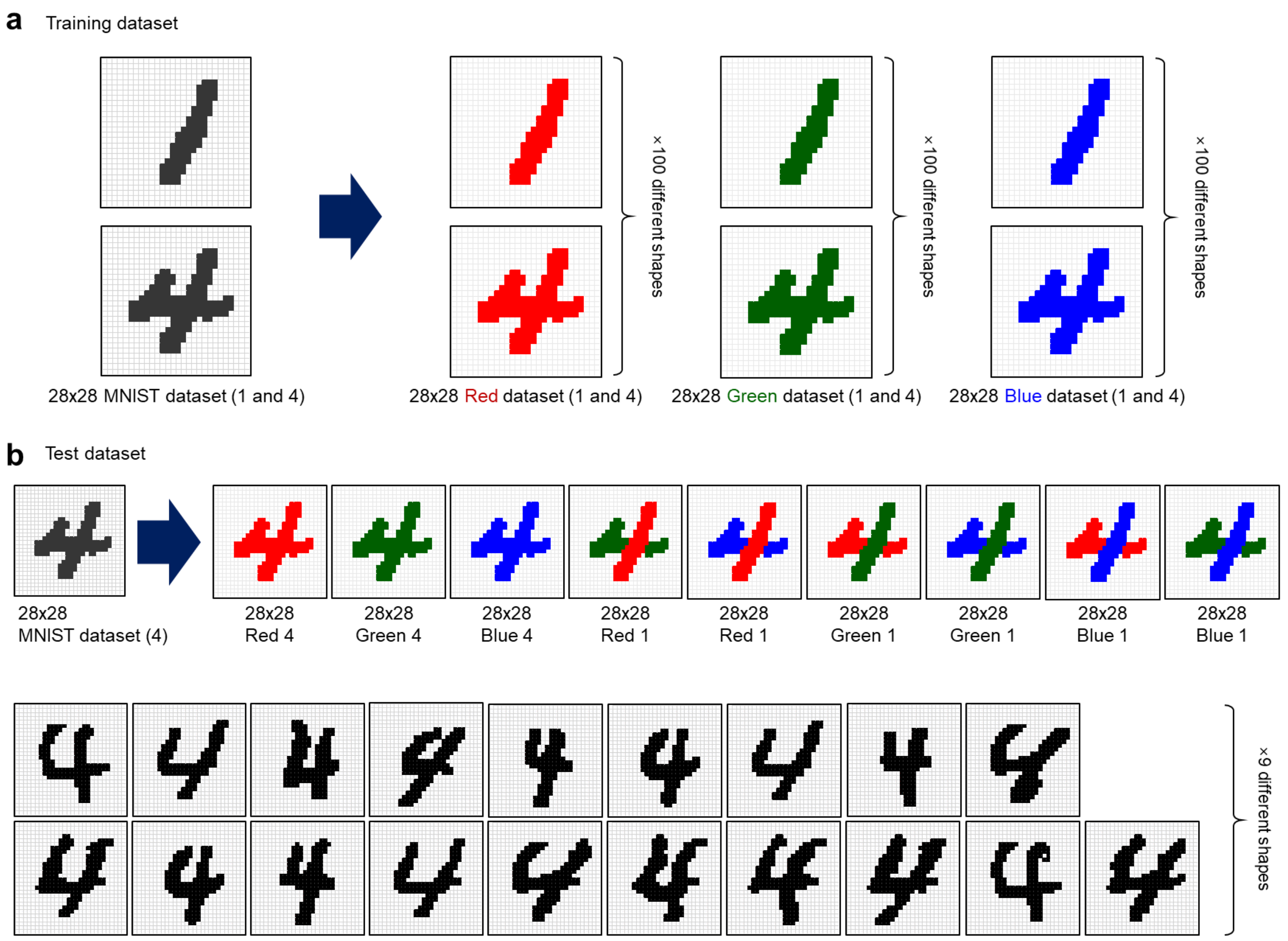


**Supplementary Figure 13 | Training & Test dataset. a,** Training dataset. Here, the MNIST dataset was used for the pattern recognition task, in which the original image size (28×28) was preserved, but a simple modification was made to generate colored numerical patterns (a total of 600 patterns). **b,** Test dataset. A simple modification was made to generate color-mixed patterns (shape of a "1" within the shape of a "4," for a total of 180 patterns).

**a**[1]

| | **This work** | | | |
|---|---|---|---|---|
| **Device type** | *h*-BN/$WSe_2$ | | | |
| **Illumination Condition** | Dark | Red | Green | Blue |
| **# of conductance state** | 100 | 100 | 100 | 100 |
| **Nonlinearity** | 1.82 (P) / -1.81 (D) | 1.89 (P) / -1.83 (D) | 1.83 (P) / -1.81 (D) | 1.83 (P) / -1.81 (D) |
| **$R_{ON}$** | 53.4 MΩ | 8.51 MΩ | 0.82 MΩ | 72.4 kΩ |
| **ON/OFF ratio** | 2.05 | 2.08 | 2.09 | 2.07 |
| **Weight increase pulse** | 0.3 V / 10 ms | 0.3 V / 10 ms | 0.3 V / 10 ms | 0.3 V / 10 ms |
| **Weight decrease pulse** | -0.3 V / 10 ms | -0.3 V / 10 ms | -0.3 V / 10 ms | -0.3 V / 10 ms |

| Cycle-to-cycle variation (σ) | < 1% | < 1% | < 1% | < 1% |
|---|---|---|---|---|

**b**[2]

| Device Type | | Energy consumption |
|---|---|---|
| **This work** | *h*-BN/$WSe_2$ | 66-532 fJ |
| **Phase change** | $Ge_2Sb_2Te_5$[3] | 2-50 pJ |
| | $Ge_2Sb_2Te_5$[4] | 121-1552 pJ |
| | Doped GeSbTe[5] | ~0.9 pJ |
| | $Ge_2Sb_2Te_5$[6] | 1 pJ |
| **Resistive change** | $TiO_x/HfO_x$[7] | 0.85-24 pJ |
| | PCMO[8] | 6-600 pJ |
| | $TiO_x$[9] | ~200 nJ |
| | $Wo_x$[10] | ~40 pJ |
| **FET-based** | Ion-doped polymer-based FET[11] | 10 pJ |
| | NOMFET[12] | ~5 μJ |
| | ENODe[13] | ~10 pJ |

**Supplementary Table 1 | Comparison of *h*-BN/$WSe_2$ synaptic device and other synaptic devices[1,2].** **a**, In order to compare *h*-BN/$WSe_2$ synaptic device with other synaptic devices reported heretofore, we extracted various parameters (weight update linearity, number of conduction states, and stability of each state) by using MLP+NeuroSim framework[1]. **b**, The energy consumption of the *h*-BN/$WSe_2$ synaptic device was also compared with the values of other synaptic devices reported heretofore[2-13].

| | $\alpha_P$ | $\alpha_D$ | $\beta_P$ | $\beta_D$ | $G_{max}$ | $G_{min}$ |
|---|---|---|---|---|---|---|
| Dark | $1.50\times10^{-10}$ | $1.50\times10^{-10}$ | 1.4 | 1.4 | $1.34\times10^{-8}$ | $6.78\times10^{-9}$ |
| Red | $1.35\times10^{-9}$ | $1.40\times10^{-9}$ | 1.5 | 1.5 | $1.17\times10^{-7}$ | $5.93\times10^{-8}$ |
| Green | $1.43\times10^{-8}$ | $1.52\times10^{-8}$ | 1.5 | 1.5 | $1.22\times10^{-6}$ | $6.12\times10^{-7}$ |
| Blue` | $1.60\times10^{-7}$ | $1.47\times10^{-7}$ | 1.5 | 1.5 | $1.38\times10^{-5}$ | $7.00\times10^{-6}$ |

**Supplementary Table 2 | Extracted Fitting Parameters for Colored-Pattern Recognition Tasks**

### Supplementary Note 1. Characterization of the optical-sensing device

We fabricated the optical-sensing device on a vdW heterostructure and analyzed the absorbance and the current driving capability at different light conditions. First, to investigate the absorbance with respect to wavelength, the ultraviolet-visible (UV-Vis) measurement was performed on the optical-sensing device. The absorbance of $WSe_2$ increased with decreasing wavelength, indicating that the number of photoexcited electron carriers increased. Then, a control voltage ($V_C$) was swept from −15 to +15 V with a fixed presynaptic voltage of −0.1 V, where we measured the current per device width (A/μm) at various light wavelengths (red, green, blue, and no light), as shown in Supplementary Figure 4. We then fixed a control voltage to be 0 V and swept the presynaptic voltage from −1 to 0 V. Without light irradiation to the device (no-light condition), our optical-sensing device showed the highest resistance of $3.33\times10^7$ Ω, while the lowest value of $1.86\times10^4$ Ω was obtained under the blue lighting condition (see Supplementary

Fig. 4c). The current per device width (A/μm) at the presynaptic node was distributed from $10^{-8}$ A/μm (no light) to $10^{-4}$ A/μm (blue), depending on the lighting condition. Supplementary Figure 4d shows photoresponsivity as a function of wavelength. As the wavelength increased from 405 to 655 nm, the photoresponsivity decreased from $7.08\times10^3$ to $6.32\times10^2$ and then to $6.19\times10^1$ A/W. Supplementary Figure 4d also shows photoresponsivity according to the power of incident light with 532 nm wavelength. As the optical power increased from 10 to 1000 pW, the photoresponsivity decreased from $4.10\times10^5$ to $3.00\times10^4$ and then to $6.70\times10^3$ A/W. Here, the photocurrents were extracted under the following bias conditions: $V_C = V_{TH}$ and $V_{Pre}$ = –0.1 or –1 V.

If the thickness of the gate dielectric layer is reduced to the level which is compatible to the current technology node, this device will exhibit optimal performance near 0 V. Moreover, the application of high-k materials will also reduce the operating voltage.

## Supplementary Note 2. Number of effective conductance states

Threshold$_{\Delta G}$ can be determined as a certain percentage of the difference between $G_{max}$ and $G_{min}$. As shown in Supplementary Figure 6a, we extracted $\Delta G$ with regard to the number of pulses with +1 V amplitude. The number of $\Delta G$ points that do not exceed threshold$_{\Delta G}$ is 36 out of 100, and thus the number of effective conductance state becomes 74 out of 100. To confirm the relationship between the number of effective conductance states and the nonlinearity, as shown in Supplementary Figure 6b, we extracted the number of effective conductance states with regard to the nonlinearity under specific values for threshold$_{\Delta G}$ (0.3%, 0.5%, 0.7%, and 0.9%). As the conductance response becomes nonlinear, the number of effective conductance states exponentially decreases. Moreover, Supplementary Figure 6b shows the value of threshold$_{\Delta G}$ to ensure the conductance states are fully effective as a function of the nonlinearity, where the threshold value decreases as the conductance response becomes more nonlinear.

## Supplementary Note 3. Nonlinearity Analysis

Generally, the hysteresis-based synaptic device is significantly affected by the nonlinearity property in both LTP and LTD curves with respect to the number of applied pulses. The nonlinear characteristics have a critical impact on recognition accuracy when the synaptic device is employed in neuromorphic computing hardware platforms such as DNN- or SNN-based neuromorphic systems[14]. Here, nonlinearity analysis was conducted for our vdW synaptic device, in light of extracting the fitting parameters that are related to the weight update formula.

Evaluation of the nonlinearity property was performed by using the following weight update formula:

$$G_{n+1} = G_n + \Delta G_P = G_n + \alpha_P e^{-\beta_P \frac{G_n - G_{min}}{G_{max} - G_{min}}} \ldots \text{ Supplementary Equation 1,}$$

$$G_{n+1} = G_n + \Delta G_D = G_n - \alpha_D e^{-\beta_D \frac{G_{max} - G_n}{G_{max} - G_{min}}} \ldots \text{ Supplementary Equation 2.}$$

Here, $G_{n+1}$ and $G_n$ denote the conductance of the synaptic device when the n+1$^{th}$ and n$^{th}$ pulses are applied. $G_{max}$ and $G_{min}$ indicate the maximum and minimum conductance values. Parameters α and β are the changing step sizes of the conductance and nonlinearity. As shown in Supplementary Figure 7a, a larger β presents greater nonlinearity. The nonlinearity values obtained from the fitted LTP and LTD curves (case 1: 0.3 V amplitude input pulse) were 1.4 and 1.4 for potentiation and depression, respectively (see Supplementary Fig. 7b). The fitting results for the LTP and LTD curves according to the input pulse amplitudes are summarized in Supplementary Table 2, where we confirmed that a greater amplitude of input pulse causes greater nonlinearity.

## Supplementary Note 4. Hysteresis Engineering

Supplementary Figure 8 shows the transfer curves of the synaptic devices fabricated on the vdW heterostructure. We swept the control voltage from −15 to 15 V and then measured the current per unit width (A/μm) between presynaptic and postsynaptic terminals. The hysteresis was not observed in the synaptic device without WCL, as shown in Supplementary Figure 8a. This is mainly because a defect-free heterojunction was created between the *h*-BN and $WSe_2$ layers. In contrast, the synaptic device with WCL clearly showed hysteresis after the 5-minute $O_2$ plasma treatment in Supplementary Figure 8b. This is attributed to the defects generated on the *h*-BN during the $O_2$ plasma treatment. This charge trapping and de-trapping mechanism through the defects leads to modulation in the carrier concentration in the $WSe_2$ channel, and finally to changing the channel conductance. We also investigated the $I_{Post}$-$V_C$ characteristic at various ambient temperatures from 170 K to 320 K, as shown in Supplementary Figure 8c. As the temperature increased, the hysteresis window was broadened. Compared to the case at room temperature, a conductance change ($\Delta G$) is predicted to be relatively small at lower temperatures.

## Supplementary Note 5. X-TEM, EELS, and EDS of the control device

Supplementary Figure 9 a, b, and c show high-resolution X-TEM, EELS, and EDS mapping images[15] of the $WSe_2$/*h*-BN interface that did not undergo the $O_2$ plasma process. Because the $O_2$ plasma treatment was not applied on the *h*-BN layer, no WCL was observed (see Supplementary Fig. 9a). In addition, as seen in Supplementary Figure 9b and c, high resolution electron energy loss spectroscopy (EELS) and

energy-dispersive X-ray spectroscopy (EDS) mapping analyses were performed to investigate the compositions of the $WSe_2$/*h*-BN interface region. In the *h*-BN regions, the O element was not observed, but B and N elements were clearly present. The $WSe_2$ regions did not show the presence of oxygen, either.

**Supplementary Note 6. Approximate uniform trap density in WCL**

In the LTP/LTD curves with a ±0.3 V amplitude pulse, as shown in Supplementary Figure 10a, a control voltage shift ($\Delta V$) for ensuring $G_{min}$ to $G_{max}$ is 1.2 V. Assuming that the $\Delta V$ is determined only by $\Delta Q_i$,

$$\Delta V = -\frac{\Delta Q_i}{C_i} = -\frac{1}{C_i}\frac{1}{t_{ox}}\int_0^{t_{ox}} x\rho \, dx = -\frac{q n_{avg}}{2C_i t_{ox}}(t_{ox}^2 - t_{bn}^2) \text{ … Supplementary Equation 3,}$$

where $n_{avg}$ is the approximate uniform trap density in the WCL layer, and $t_{ox}$ is $t_{ox} = t_{bn} + t_{wcl}$ ($t_{bn}$ and $t_{wcl}$ are the thicknesses of *h*-BN and WCL). $C_i$ in equation S11a is defined as $C_{bn}||C_{wcl} = \frac{C_{bn}C_{wcl}}{(C_{bn}+C_{wcl})} = \frac{\varepsilon_{bn}\varepsilon_{wcl}}{(\varepsilon_{bn}t_{wcl}+\varepsilon_{wcl}t_{bn})}$, where $C_{bn}$ and $C_{wcl}$ are capacitances for *h*-BN and WCL, and $\varepsilon_{bn}$ and $\varepsilon_{wcl}$ mean the permittivity values for *h*-BN and WCL.

Therefore, $n_{avg}$ is obtained by Supplementary Equation 3 as

$$n_{avg} = \frac{-2C_i t_{ox}\Delta V}{q(t_{ox}^2 - t_{bn}^2)} \text{ … Supplementary Equation 4,}$$

which is approximately $7.4\times10^{17}$ $cm^{-3}$ when assuming $\varepsilon_{bn} = 4.5\varepsilon_0$ and $\varepsilon_{wcl} = 2.32\varepsilon_0$ ($t_{bn}$ = 23 nm, $t_{wcl}$ = 10.9 nm).

**Supplementary Note 7. Post-Synaptic Currents**

We analyzed the post-synaptic current characteristics of our vdW synaptic device under diverse activation pulse conditions. For the EPSC, we applied pulses with a width of 10 ms and an amplitude of +2 V to the SCT. For the case of the inhibitory current (IPSC), a pulse with the same width and a negative amplitude of −2 V was applied. Supplementary Figure 11a shows the conductance trajectories for the EPSC and IPSC. After applying the pulses at t = 1 s with a width of 10 ms, the conductance trajectories reach $\Delta G$ values of +50% and −45% for EPSC and IPSC, respectively. We also investigated the dependence of the $\Delta G$ on the pulse amplitude and duration. The higher the amplitude of the pulse with the fixed pulse duration (10 ms for this analysis), the greater the increase in $\Delta G$ for both EPSC and IPSC, as shown at Supplementary Figure 11b. A similar trend was also observed according to a change in pulse

duration from 5 ms to 50 ms (here, pulse amplitude was 1 V), and the results are shown at Supplementary Figure 11b.

## Supplementary References


1. Chen, P.-Y., Peng, X., Yu, S. NeuroSim+: An integrated device-to-algorithm framework for benchmarking synaptic devices and array architectures. *IEEE Int. Electron Devices Meeting (IEDM)*. (2017).

2. Kuzum, D., Yu, S. & Wong, H. S. P. Synaptic electronics: materials, devices and applications. *Nanotechnology*. **24**, 1-22 (2013).

3. Kuzum, D., Jeyasingh, R. G. D., Yu, S. & Wong, H. S. P. Low-Energy Robust Neuromorphic Computation Using Synaptic Devices. *IEEE. Trans.* **59**, 3489-3494 (2012).

4. Suri, M. *et al*. Phase Change Memory as Synapse for Ultra-Dense Neuromorphic Systems: Application to Complex Visual Pattern Extraction. *IEEE Int. Electron Devices Meeting (IEDM)*. pp 4.4.1-4 (2011).

5. Kim, S. *et al*. NVM neuromorphic core with 64 k-cell (256-by-256) phase change memory synaptic array with on-chip neuron circuits for continuous in-situ learning. *IEEE Int Electron Devices Meeting (IEDM).* (2015).

6. Ambrogio, S. *et al*. Unsupervised learning by spike timing dependent plasticity in phase change memory (PCM) synapses. *Frontiers in Neuroscience.* **10** (2016).

7. Yu, S., Gao, B., Fang, Z., Kang, J. & Wong, H. S. P. A neuromorphic visual system using rram synaptic devices with sub-pJ energy and tolerance to variability: experimental characterization and large-scale modeling. *IEEE Int. Electron Devices Meeting (IEDM)*. pp 10.4.1-4 (2012).

8. Park, S. *et al*. Neuromorphic speech systems using advanced ReRAM-based synapse. *IEEE International Electron Device Meeting (IEDM)*. **625-628** (2013).

9. Seo, K. *et al*. Analog memory and spike-timing-dependent plasticity characteristics of a nanoscale titanium oxide bilayer resistive switching device. *Nanotechnology*. **22**, 254023 (2011).

10. Yang, R. *et al*. On-demand nanodevice with electrical and neuromorphic multifunction realized by local ion migration. *ACS Nano*. **6**, 9515-9521 (2012).

11. Lai, Q. *et al*. Ionic/electronic hybrid materials integrated in a synaptic transistor with signal processing and learning functions. *Adv. mat.* **22**, 2448-2453 (2010).

12. Alibart, F. et al. An organic nanoparticle transistor behaving as a biological spiking synapse. *Adv. funct. mat*. **20**, 330-337 (2010).

13. Burgt, Y. v. d. et al. A non-volatile organic electrochemical device as a low-voltage artificial synapse for neuromorphic computing. *Nat. mater*. **4856**, 1-6 (2017).

14. Park, S. *et al*. Electronic system with memristive synapses for pattern recognition. *Sci. Rep*. **5**, 10123 (2015).

15. Kim, S. M. *et al*. Synthesis of large-area multilayer hexagonal boron nitride for high materials performance. *Nat. Commn*. **9662** (2015).
